# Deficits in Letter-Speech Sound Associations but Intact Visual Conflict Processing in Dyslexia: Results from a Novel ERP-Paradigm

**DOI:** 10.3389/fnhum.2017.00116

**Published:** 2017-03-09

**Authors:** Sarolta Bakos, Karin Landerl, Jürgen Bartling, Gerd Schulte-Körne, Kristina Moll

**Affiliations:** ^1^Department of Child and Adolescent Psychiatry and Psychotherapy, University Hospital MunichMunich, Germany; ^2^Institute of Psychology, University of GrazGraz, Austria

**Keywords:** dyslexia, letter-speech sound associations, visual conflict processing, ERP, N1, N2, conflict slow potential

## Abstract

The reading and spelling deficits characteristic of developmental dyslexia (dyslexia) have been related to problems in phonological processing and in learning associations between letters and speech-sounds. Even when children with dyslexia have learned the letters and their corresponding speech sounds, letter-speech sound associations might still be less automatized compared to children with age-adequate literacy skills. In order to examine automaticity in letter-speech sound associations and to overcome some of the disadvantages associated with the frequently used visual-auditory oddball paradigm, we developed a novel electrophysiological letter-speech sound interference paradigm. This letter-speech sound interference paradigm was applied in a group of 9-year-old children with dyslexia (*n* = 36) and a group of typically developing (TD) children of similar age (*n* = 37). Participants had to indicate whether two letters look visually the same. In the incongruent condition (e.g., the letter pair A-a) there was a conflict between the visual information and the automatically activated phonological information; although the visual appearance of the two letters is different, they are both associated with the same speech sound. This conflict resulted in slower response times (RTs) in the incongruent than in the congruent (e.g., the letter pair A-e) condition. Furthermore, in the TD control group, the conflict resulted in fast and strong event-related potential (ERP) effects reflected in less negative N1 amplitudes and more positive conflict slow potentials (cSP) in the incongruent than in the congruent condition. However, the dyslexic group did not show any conflict-related ERP effects, implying that letter-speech sound associations are less automatized in this group. Furthermore, we examined general visual conflict processing in a control visual interference task, using false fonts. The conflict in this experiment was based purely on the visual similarity of the presented objects. Visual conflict resulted in slower RTs, less negative N2 amplitudes and more positive cSP in both groups. Thus, on a general, basic level, visual conflict processing does not seem to be affected in children with dyslexia.

## Introduction

The ability to read is very fundamental in our daily life, allowing the transfer of knowledge and information. It is not only important for an individual’s academic and professional carrier, but is crucial for a successful integration into a modern society. Thus, difficulties in the acquisition of reading skills can have a negative impact on several aspects of life. This might be the case in developmental dyslexia (dyslexia), a developmental disorder affecting approximately 5–11% of the population (Jones et al., 2016). Children and adults with dyslexia have difficulties in accurate or fluent reading and accurate spelling, despite adequate schooling, intelligence, and intact sensory abilities (Lyon et al., 2003; for a review see Peterson and Pennington, 2012).

One of the most important prerequisites of reading in alphabetic orthographies is the build-up of letter-speech sound associations (Ehri, 2008). Longitudinal studies indicate that letter-sound knowledge is a strong predictor of later literacy skills (Lonigan et al., 2000; Schatschneider et al., 2004; Caravolas et al., 2012). Children with a familial risk for dyslexia take longer to learn the associations between letters and speech-sounds than control children (Torppa et al., 2006), and the build-up of associations might be less automatic (Bishop, 2007). Even though there are no differences between good and poor readers in their behaviorally measured letter knowledge (i.e., knowing the letter and the corresponding sound) (Stein and Walsh, 1997; Facoetti et al., 2001), neurophysiological studies suggest that automatic integration of letters and speech-sounds (i.e., immediate activation of the speech sound by the sight of a letter) might be a problem in dyslexia not only in early but even in adult years (Blomert, 2011; Froyen et al., 2011). Automated letter-speech sound associations are likely to play a crucial role for fluent reading given that fluent reading requires fast access from the visually presented letter or word to its phonological form. Thus, understanding the developmental differences in the build-up of automated letter-speech sound associations between children with dyslexia and typically developing (TD) children might help to identify and better understand the problems which may cause difficulties in reading acquisition. For this reason, we examined the strength of letter-speech sound associations in children with dyslexia and TD children in a newly developed event-related potential (ERP) paradigm.

Until now, automatization of letter-speech sound associations has been commonly examined applying a visual-auditory passive oddball paradigm during ERP measurement (Froyen et al., 2008; Moll et al., 2016). In this visual-auditory oddball paradigm, frequent standard congruent letter-sound pairs (e.g., the letter “a” with the sound /a/) are compared to rare deviant incongruent letter-sound pairs (e.g., the letter “a” with the sound /o/). The measured ERP component derived from the continuous electroencephalogram (EEG) is called audiovisual mismatch-negativity (audiovisual MMN). The audiovisual MMN is measured as a difference wave, built from the difference between the standard and deviant condition, reflecting the strength of letter-speech sound integration. Enhanced negativity in the deviant (/o/-sound) condition is caused not only by the deviation of the /o/-speech sound from the standard /a/-speech sound, but further strengthened by the dissociation between the presented /o/-speech sound and the standard letter “a” (Froyen et al., 2008, 2009, 2011; Žarić et al., 2015). However, this visual-auditory oddball paradigm has also some disadvantages (for a review see, Bishop, 2007). It is a passive paradigm, thus behavioral correlates of the neurophysiological differences cannot be assessed. This might be problematic, especially when assessing children and clinical populations. The experimental setup of the visual-auditory passive oddball paradigm requires a relatively high stimulus-repetition rate, which might lead to problems in maintaining attention in these populations. Without behavioral measurements as a control for task performance, it can be questioned whether the participants correctly processed the task. Furthermore, the audiovisual MMN component is measured as a difference waveform between the standard and deviant condition, which results in a lower signal-to-noise ratio than measuring the original ERP waveforms. It has been shown that the test-retest reliability of the audiovisual MMN is lower than the reliability of the standard peaks (e.g., P1, N1, P2) (McArthur et al., 2003). Lastly, the experimental situation of seeing the letter and hearing the speech sound at the same time does not reflect the silent reading situation experienced in everyday life, which can compromise external validity. In real life reading situations, children are only seeing the letters but not hearing the speech sounds.

Thus, we developed a new neurophysiological paradigm to measure automatization strength using the theoretical framework of the classical Stroop-paradigm (Stroop, 1935) and the letter-matching paradigm described by Posner and Mitchell (1967), which we named letter-speech sound interference paradigm. In the classical Stroop-paradigm, words for colors are presented in different colors. In the incongruent condition, the color of the word and the meaning of the word is not the same (e.g., the word red written in green), whereas in the congruent condition both the meaning and the color of the word are the same (e.g., the word red is written in red). Participants have to respond to the color of the word presented and ignore the meaning of the word. In fluent readers, response times (RTs) are typically slower in the incongruent than in the congruent condition. This effect is called the Stroop-interference effect (for a review see MacLeod, 1991). The interference effect is not present in non-readers (Schadler and Thissen, 1981), but starts to increase with the progress of reading instruction, when reading becomes more automatized and fluent (Peru et al., 2006). Thus, the Stroop-interference effect can be explained by the automatic activation of the task-irrelevant information (in this case the word meaning), which is in conflict with the task-relevant information (the color of the word) and slows RTs. The size of the interference effect is therefore an indicator of the conflict between the task-irrelevant and task-relevant information and thus reflects the degree of reading automatization.

In our letter-speech sound interference paradigm, the task of the participant is to indicate whether two letters look exactly the same, irrespective of which phoneme they represent. This idea is similar to the letter matching paradigm, first implemented by Posner and Mitchell (1967). However, until now, the letter-matching paradigm was mainly used to analyze visual recognition and memory retrieval processes. To our best knowledge, we are the first to apply the letter-matching paradigm in the context of letter-speech sound associations and within the theoretical framework of an interference effect using ERP measurements.

In analogy to the Stroop-studies, we assume that the participants in our letter-speech sound interference paradigm have two concurrent sources of information: their task is to decide whether two visually presented letters look physically the same. The task-relevant information thereby is the visual appearance of the letters (e.g., the visual characteristics of “A” or “a”), whereas the information about the associated speech-sound (e.g., the phoneme /a/), which is supposed to get automatically activated, is irrelevant. An incongruent trial in this case would be, for example, the letter-pair A-a, because the visual appearance of the letters is not the same, even though they can be assigned to the same phoneme /a/. Thus, the visual task-relevant information is in favor of a “no” (they are not the same) response, but the automatically activated phonological information suggest a “yes” (they are the same) response. In a congruent trial, like for example the letter-pair A-e, both the visual appearance and the associated speech sound of the letters are different, meaning that both the visual information and the automatically activated phonological information suggest a “no” (they are not the same) response. Letter-speech sound associations are highly automatic in advanced readers (Froyen et al., 2009), thus speech sounds associated with the letters are expected to get automatically activated resulting in an interference effect in the incongruent condition. This would lead to slower RTs in the incongruent (e.g., A-a) than in the congruent (e.g., A-e) condition. In the German orthography, where letter-speech sound correspondences are fairly consistent, this effect is expected to be especially strong.

The size of the reaction time difference and the difference between the ERP components of the incongruent and congruent conditions is expected to give us insights about letter-speech sound automatization strength. Even if there are no differences between good and poor readers in their behaviorally measured letter-sound knowledge (Froyen et al., 2011), we might be able to find group differences when we measure letter-speech sound automatization strength, rather than letter knowledge and apply more sensitive methods, like EEG, rather than only behavioral measurements.

However, since the implemented neurophysiological letter-speech sound interference paradigm is entirely new, we have no reference studies as to what ERP components to examine, but we assume that the conflict in the incongruent condition would lead to similar ERP-effects than other conflict related paradigms like the Stroop- and the flanker task. Based on this literature, we decided to focus our analysis on three classical, conflict-related ERP components. We analyze two early components, the N1 and the N2, and one late component, the conflict slow potential (cSP).

The fronto-central N1 has been shown to be sensitive to conflict in Stroop-, and flanker-task experiments applying either visual (Johnstone et al., 2009) or auditory stimuli (Näätänen and Picton, 1987; Henkin et al., 2010; Yu et al., 2015). It is commonly measured over fronto-central sites as the most negative deflection between 100 and 200 ms after stimulus presentation and has been associated with conflict detection (Yu et al., 2015). The results of auditory Stroop experiments suggest that fronto-central N1 amplitudes are less negative in incongruent than in congruent trials (Lew et al., 1997; Henkin et al., 2010; Yu et al., 2015); however, there is also a study which found the opposite pattern in a visual flanker task (Johnstone et al., 2009). Thus, the direction of the conflict-related N1 amplitude modification appears to depend on the characteristics of the applied stimuli and paradigm.

The fronto-central N2, commonly observed as a negative deflection peaking approximately 250–350 ms after stimulus onset, reflects conflict detection and conflict monitoring processes mediated by the anterior cingulate cortex (for a review see, Larson et al., 2014). Depending on the paradigm, the N2 amplitude is either more negative in incongruent than in congruent trials (van Veen and Carter, 2002a,b; Yeung et al., 2004; Johnstone et al., 2009) or more negative in congruent than in incongruent trials (Yu et al., 2015). However, there is also a study, which did not find any effects of conflict on the N2 amplitudes (Henkin et al., 2010). Thus, the existing literature is inconclusive about the occurrence and directionality of the conflict-related N2 amplitude effect.

The cSP is a sustained positivity beginning approximately 500 ms after stimulus presentation. Over centro-parietal sites, the cSP has been found to be more positive on incongruent trials than on congruent trials (Liotti et al., 2000; Larson et al., 2009; Donohue et al., 2012; Yu et al., 2015). It has been commonly interpreted as reflecting conflict resolution (West and Alain, 2000; West, 2003) or response selection processes (West et al., 2005). The neural generators of the cSP are most probably the lateral frontal and posterior cortices (West, 2003; Hanslmayr et al., 2008; for a review see Larson et al., 2014).

The effect of conflict on these three ERP might, however, be different in individuals with developmental dyslexia. Mahé et al. (2014) for example, found reversed N1 and missing N2 effects in a group of adults with dyslexia when compared to typical readers in a flanker task comprising congruent and incongruent trials. Thus, in order to ensure that the congruency-related effects of our letter-speech sound interference experiment reflect differences in letter-speech sound association strength and not only general differences in conflict processing between the groups, we implemented a control task, which we named visual interference experiment. Instead of real letters, we used false fonts, which were visually similar to the letters. The conflict in the incongruent condition of this experiment is based purely on the visual characteristics of the stimuli because there are no speech sounds associated with the false fonts. The task of the participants was to indicate whether two false fonts looked exactly the same. In the incongruent condition the same fonts were presented in different sizes, whereas in the congruent condition two different false fonts were displayed. The conflict in this visual interference experiment is thus based on the visual similarity of the stimuli, whereas the conflict in the letter-speech sound interference experiment is caused by the automatic activation of the letter-related speech sounds.

Taken together, our study comprising the letter-speech sound interference and the visual interference experiment is designed to answer the following questions:

(1) Do children with dyslexia show impairments in the automatization of letter-speech sound associations when compared to TD 9-year-olds? The difference from previous investigations of this question is the application of single letter-stimuli, without the synchronous presentation of auditory stimuli. Thus, our paradigm might be more closely related to the everyday situation of silent reading than the synchronous presentation of letters and sounds. This might increase external validity. Furthermore, the opportunity to assess individual task performance might help to control for motivational and attentional problems and to increase the reliability of the measurement.
(2) Is the time point of speech-sound activations by the sight of letters delayed in dyslexia? The combination of our paradigm with the high temporal resolution of ERP-measurements can help us to answer the question, at which time point speech sounds associated with letters get activated. This information could help us to shed more light on the causes of dysfluent reading in dyslexia.
(3) Are conflict-processing difficulties in dyslexia limited to specific, language-related domains, or do they extend in other domains? Dyslexia-related conflict processing literature is very sparse. At the moment, the only ERP-study examining conflict processing in dyslexia is the study of Mahé et al. (2014). However, their study implemented a type of flanker task, which examines a special aspect of visual conflict control, focusing on the suppression of flankers. Visual processing deficits may not be as strongly related to the core symptoms of dyslexia as language-related deficits, thus it might be difficult to relate the findings in this domain to the specific reading difficulties in dyslexia. Our letter-speech sound interference experiment extends the dyslexia-related conflict processing literature into a more language-related domain.
(4) Are there general differences between TD children and children with dyslexia in processing of visual stimuli? Deficits in general visual processing and visual attention are frequently assumed in dyslexia (Facoetti and Turatto, 2000; Stein, 2014), however, this question is still severely discussed (Wimmer and Schurz, 2010). The visual interference control experiment allowed us to examine whether there are general visual processing deficits in dyslexia or whether the reported deficits are limited to specific domains.

As the conflict in the letter-speech sound interference experiment is based on the automatic activation of letter-speech sound associations, which are expected to be strong and highly automatic in TD German 9-year-olds, but impaired in children with dyslexia (Moll et al., 2016), we expect to find different congruency-related effects in the control and the affected group. In the control group of TD children, we expect to see fast and strong conflict-related effects, reflected in less negative N1 and N2 amplitudes in the incongruent than in the congruent condition. In previous studies, the direction of the conflict-related N1 and N2 amplitude modification was somewhat inconsistent; however, it seems that in auditory paradigms, the conflict reduces amplitudes (Henkin et al., 2010; Yu et al., 2015). Our conflict depends on the activation of auditory information, thus, we expect our paradigm to be more strongly related to the findings of auditory- than visual studies. The cSP is expected to be more positive in the incongruent than in the congruent condition (Yu et al., 2015). Behaviorally, we expect slower RTs in the incongruent than in the congruent condition, reflecting a strong interference effect, at least in the control group. In the group of children with dyslexia, however, letter-speech sound associations might be weak and impaired, as shown in previous ERP-studies (Schulte-Körne et al., 1998; Bishop, 2007; Moll et al., 2016). Thus, speech sounds associated with the letters might not get automatically activated, resulting in less interference and weak or absent conflict-related RT- and ERP-effects.

In the visual interference control experiment, the conflict in the incongruent condition is based on visual aspects of the stimuli. Again, RTs are expected to be slower in the incongruent than in the congruent condition, besides more positive cSP amplitudes in the incongruent compared to the congruent condition. In most visual studies, the N1 and N2 amplitudes are enlarged by visual conflict, thus we expect to find more negative N1 and N2 amplitudes in the incongruent condition. However, whether these effects are comparable between the groups remains unclear. The conflict in the incongruent condition of this experiment is based on visual aspects of the stimuli, thus, we might find similar effects in the groups as visual processing is most probably intact in dyslexia (Wimmer and Schurz, 2010). However, there is also evidence for impaired visual attention in dyslexia (Stein and Walsh, 1997; Facoetti et al., 2003).

## Materials and Methods

### Participants

Participants were selected and recruited in a two-stage selection process based both on classroom screening and individual testing. In the first step, children were selected based on an extensive classroom screening with 1488 children at the end of the 3rd grade. The screening was carried out in 46 primary schools in the rural and urban areas of Munich (Germany). Reading fluency and spelling were assessed by standardized classroom tests (SLS 2–9: Wimmer and Mayringer, 2014; DRT-3: Müller, 2004). Children were classified as reading and spelling impaired (dyslexic group) if they scored at or below the 18th percentile on the reading test and below the 20th percentile on the spelling test. Children with reading and spelling performances between the 25th and 75th percentile were included in the control group.

In addition, a classroom test measuring non-verbal IQ (CFT 20-R: Weiß, 2006) was administered. Only children with a non-verbal IQ ≥ 85 were invited for further testings. Further inclusion criteria were German as 1st language, normal or corrected-to-normal vision, absence of neurological deficits and no symptoms of AD(H)D as measured by a standardized questionnaire answered by caregivers (DISYPS-II: Döpfner et al., 2008). All children fulfilling criteria for the dyslexic group were invited for individual testing. For the control group, we invited gender and IQ-matched children from the same classroom and school. Altogether, 163 children (87 control children and 76 children with dyslexia) were invited to individual testings (estimated prevalence rate of 5.11%).

From the invited 163 children plus 1 volunteer (based on word-of-mouth recommendation), 85 children (42 control children and 43 children with dyslexia) gave written consent and took part in the study. Before inclusion into the final sample, reading scores were verified by an individually administered 1-min word and pseudoword reading fluency test (SLRT-II: Moll and Landerl, 2010) in a second selection step. Participants were only included in the study if their reading performance measured by the SLRT-II reflected their reading performance in the screening test (SLS 2-9). In order to be included in the dyslexic group, children had to score below the 18th percentile on at least one subtest of the SLRT-II (word- or pseudoword reading). Children in the control group had to score above the 20th percentile for both subtests.

Altogether, we had to exclude five children from the control group (two children based on their reading scores and three children based on their EEG data including one child who misunderstood the task, one child who had incomplete EEG data and one child who did not have enough artifacts-free ERP segments) and seven children from the dyslexic group (three children based on their high reading scores above the cutoff, and four children based on their EEG data including one child who misunderstood the task, one child who had incomplete EEG data and two children who did not have enough artifacts-free ERP segments).

This resulted in a final overall sample size of 37 children in the control group and 36 children in the dyslexic group. There were no significant differences between the groups in age, intelligence, gender, or handedness (all *p*s > 0.49; see **Table 1**). However, in line with our selection criteria, the groups differed in reading speed and spelling performance (all *p*s < 0.001; see **Table 1**).

**Table 1:** Descriptive statistics of the groups.

	Control group (*n* = 37)		Dyslexic group (*n* = 36)		*t*-value	*p*-value
	*M*	*SD*	*M*	*SD*		
Age in years	9.47	0.32	9.50	0.50	0.37	0.71^1^
IQ	110.57	10.59	109.14	13.38	-0.51	0.61^1^
ADHD questionnaire	0.42	0.29	0.50	0.30	1.14	0.26^1^
Handedness (right/left)	32/5		33/3			0.71^2^
Gender (males/females)	20/17		16/20			0.49^2^
Reading speed	52.05	12.85	10.19	8.75	-14.90	0.00^1^
Spelling	57.46	11.82	9.94	6.16	-21.61	0.00^1^
SLRT-II words	54.15	17.35	7.28	6.40	-15.39	0.00^1^
SLRT-II pseudowords	53.60	19.57	12.17	8.58	-11.77	0.00^1^

*Means (M) and standard deviations (SD) are reported separately for the groups. IQ is based on the CFT-20-R. The total value of the DYSIPS-II ADHD questionnaire is reported (combined for the three scales; inattention, hyperactivity, and impulsivity). Handedness was estimated by self-report. Reading speed and spelling scores are based on the SLS 2-9 and the DRT-3/DRT-4, respectively and are reported in percentile ranks. SLRT-II scores are reported separately for word- and pseudoword- reading and are measured in percentile ranks.*

*^1^*t*-test for independent samples.*

*^2^Pearson’s chi-square test.*

The study was approved by the Institutional Review Board of Medical Faculty of the University Hospital Munich and was performed in accordance with the latest version of the Declaration of Helsinki and in compliance with national legislation. Parents and children were informed in detail about the experimental procedures and the aims of the study, and gave their written consent prior to inclusion in the study. Children received vouchers in return for their participation.

### Behavioral Measurements

#### Screening in Classroom Settings

The screening took place in classroom settings within a 3 month time period at the end of grade 3. Reading, spelling and IQ-tests (SLS 2-9, DRT-3 and CFT-20-R) were administered by trained research assistants.

#### Reading

In the classroom administered reading fluency test (SLS 2-9: Wimmer and Mayringer, 2014; parallel-test reliability *r* = 0.95 and content validity *r* = 0.89 for grade 2) children were asked to read sentences silently and to mark them as semantically correct or incorrect (e.g., “Trees can speak”). After 3 min, the task was terminated and at evaluation, the number of correctly marked sentences was calculated.

#### Spelling

Spelling was assessed using a standardized classroom test (DRT-3: Müller, 2004; parallel-test reliability *r* = 0.92 and content validity *r* = 0.78). The task consisted of 44 single words which had to be written into sentence frames. The examiner first dictated the word, then read the full sentence, and repeated the dictated word. The number of correctly spelled words was scored. One participant with dyslexia did not take part in the screening but volunteered for participation during the individual testing phase at the beginning of grade 4 (see Participants). The screening measure was, therefore, adapted for this child and spelling was assessed by the corresponding version of the test for grade 4 (DRT-4: Grund et al., 2004; split-half reliability *r* = 0.92 and content validity *r* = 0.68–0.94).

#### CFT-20-R

The German version of the Culture Fair Intelligence Test (CFT 20-R; Weiß, 2006) was administered in order to estimate non-verbal cognitive abilities of the participants without the influence of sociocultural and environmental factors. The test consists of four subtests: Series, Classification, Matrices, and Topology and has a high reliability (*r* = 0.92–0.96) and construct validity (correlation with the “g”-factor *r* = 0.78–0.83).

#### Individual Assessments

Individual testing was part of a large cognitive and neurophysiological test battery and was divided into three testing sessions on two or three different days. The maximum time interval between the behavioral assessment and the EEG measurement was 96 days (mean: 20.56 days).

#### Word- and Pseudoword Reading

An individually administered 1-min word and pseudoword reading fluency test (SLRT-II; Moll and Landerl, 2010; parallel-test reliability *r* = 0.90–0.94 and content validity *r* = 0.69–0.85 for grade 3) was used. The test contains a word and pseudoword reading list with items increasing in length and complexity. Children were asked to read each list aloud as fast as possible without making any errors. The relevant measure is the number of correctly read words and pseudowords read within the 1 min time limit.

#### DYSIPS-II

In order to exclude children with ADHD and estimate attentional problems in our participants, we conducted a short telephone interview with one of the participant’s caregiver based on the ADHD questionnaire of the DISYPS-II (Döpfner et al., 2008). The DYSIPS-II is a well-established standardized structured interview for psychiatric disorders in children and adolescents based on DSM-IV and ICD-10 guidelines (Cronbach’s α = 0.87–0.94 for parental ratings of ADHD symptoms). The ADHD-questionnaire comprises of 20 questions corresponding to the three main dimensions of the ADHD symptomology: attentional deficits, hyperactivity, and impulsivity.

### ERP Paradigm and Procedure

**FIGURE 1:**
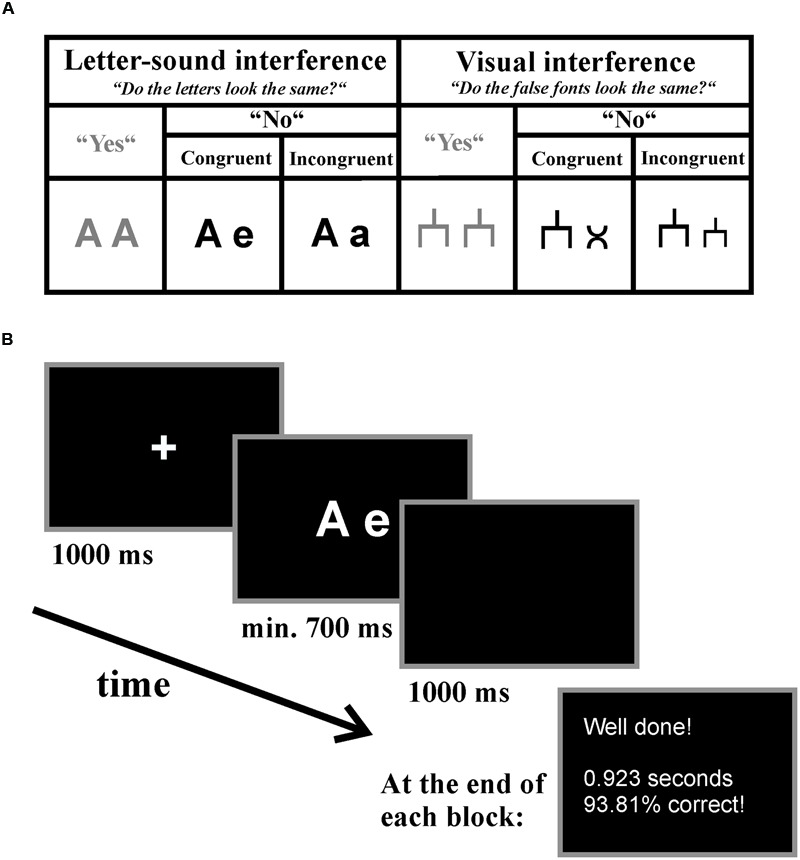
**Letter-speech sound interference and visual interference task. (A)** Experimental conditions and an example of the presented stimuli in each condition listed separately for the tasks. **(B)** An example trial of the letter-speech sound interference experiment. Participants were instructed to decide via button-press whether the presented stimuli looked visually the same and received feedback about their general performance at the end of each block.

During EEG acquisition, children performed two novel experiments, which we named letter-speech sound interference and visual interference task. The experiments were presented block-wise and the presentation order was counterbalanced between participants. The child’s task was to indicate by button press whether the visually presented stimuli (pairs of letters or pairs of false fonts) looked exactly the same. There were three experimental conditions in both experiments: (1) the incongruent (e.g., different visual appearance but same phoneme: A-a) and (2) the congruent (e.g., different visual appearance and different phoneme: A-e) condition, which both required a “no”-answer, and (3) the “yes”-answer (e.g., A-A) condition (see **Figure 1**). However, the “yes”-answer condition was only introduced in order to have two answer options resulting in a meaningful task, but it did not bear any theoretical importance. Thus, main analyses were carried out only for the congruent and incongruent “no”-answer conditions but not for the “yes”-answer condition. For the “yes”-answer condition, we report descriptive data only.

In the letter-speech sound interference experiment we used 10 letters (A, B, D, E, F, H, M, N, R, and T) written either in upper or in lower case to build the letter-pairs. These letters were selected because they have visually distinct upper- and lower-case forms (as compared for example to C or K). In the incongruent condition (e.g., A-a), the same letters written once in upper and once in lower case were presented. In this condition, the speech sounds corresponding to the two presented letters were the same, but the visual appearance of the two letters were different. Thus, we expected a conflict between the automatically activated, task irrelevant information (associated speech sounds) and the task relevant information (visual appearance of the letters). In the congruent condition (e.g., A-e), two different letters were presented. Thus, both the speech-sounds associated with the two presented letters (automatically activated, but task irrelevant information) and the visual forms of the two presented letters (the task relevant information) were different, and implied the same answer. In the yes-answer condition (e.g., A-A), the same letters written in the same case were presented (see **Figure 1**).

This procedure resulted in 10 possible letter-pairs in the incongruent condition. In order to keep the visual effects balanced, the letter-pairs were presented both with upper-case to the left (e.g., A-a, M-m) and with lower case to the left (e.g., a-A, m-M) versions, resulting in 20 different stimulus pairs. Each of these stimulus pairs was repeated three times throughout the experiment, thus the incongruent condition consisted of 60 trials altogether. In order to keep the conditions comparable, we chose 10 letter pairs for the congruent condition (out of the 45 possible combinations). In selecting these letter-pairs, we avoided letter-combinations which could have resembled meaningful abbreviations in German and the combination of consonants and vowels, in order to avoid easily pronounceable combinations. These 10 selected letter-pairs comprised one upper- and one lower-case letter, and were presented similarly to the incongruent condition also in their forward (e.g., T-f, A-e) and reversed version (e.g., f-T, e-A). Again, the stimulus pairs were repeated three times, which resulted in a total amount of 60 congruent-trials. Thus, the total amount of trials and the amount of stimulus-repetitions was the same in the incongruent (e.g., A-a) and in the congruent (e.g., A-e) condition. There were 90 trials in the yes-answer condition, 45 of them presented in lower case (e.g., a-a, m-m) 45 of them presented in upper case (e.g., A-A, M-M), which resulted in an average repetition rate of 4.5 of each yes-answer stimulus. The stimulus-repetition rate of this condition was thus somewhat higher than the repetition rate of the congruent and incongruent conditions, and the ratio of “yes” and “no” answers were 90–120, but since the “yes”-answer condition was not included in the later analysis, the differences between the conditions were not expected to influence the results.

Incongruent, congruent and yes-answer trials were presented in four pseudorandomized lists in an intermixed manner. The pseudorandomization ensured that no more than four consecutive trials had the same answer (“yes” or “no”), preventing tendencies to automatic responses. The four pseudorandomized lists were randomly assigned to the participants within each group. To ensure that the participants understood the task, the experiment was preceded by a short practice block (consisting of eight trials; two congruent, two incongruent, and four “yes”-answer trials). Each experiment was divided into two blocks with a short break in between, thus the whole processing comprised four blocks (two blocks in the letter-speech sound interference and two blocks in the visual interference experiment). One block comprised 105 stimuli and lasted 5 min, thus the whole procedure took approximately 20 min. After each break, and at the end of the experiment, participants received feedback about their general performance in the present block (percentage of correct answers and response speed in ms).

In the visual interference control experiment we assigned a false font (for an example see **Figure 1**) to each letter, thus we used 10 different false fonts presented either in the relatively big (equivalent to the upper-case letters) or in the relatively small size (equivalent to the lower-case letters) to build the false font-pairs. In the congruent condition, two different fonts were presented. In the incongruent condition, the same fonts were presented in big and small sizes. Thus, in this condition there was a visual conflict based on the visual similarity of the objects. Again, in the “yes”-answer condition, the same false fonts in the same size were presented (see **Figure 1**).

All stimuli (both letters and false fonts) were presented in white on black background in the center of a 24 inches monitor with a refresh rate of 60 Hz and a high resolution of 1920 × 1080 using E-Prime^®^ 2.0 software (Psychology Software Tools, Inc.). The computer screen was placed 70 cm in front of the children which resulted in a vertical visual angle of 1.03–1.38° and in a horizontal visual angle of 2.20–3.90° depending on the presented stimuli. Each trial started with a fixation cross (Arial, 52, bold) which remained on the screen for 1000 ms. Afterward, the stimulus pairs appeared (letters: Arial, 52, bold). Children were instructed to respond by pressing the right button for “yes” if the stimuli looked visually the same and the left button for “no” if the stimuli did not look the same on a two-key keyboard as fast as possible. The stimuli remained on the screen for at least 700 ms or until response in case it took longer. The next trial started after a 1000 ms-long blank screen. At the end of each block participants received feedback about their performance (see **Figure 1**).

### ERP Recording and Analysis

**FIGURE 2:**
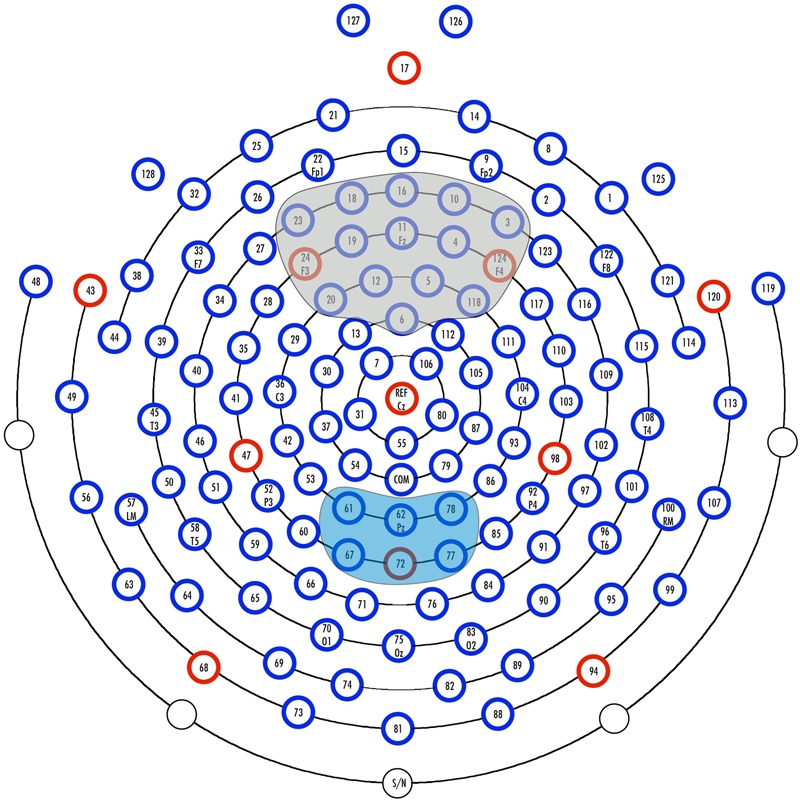
**Illustration of the 128-channel system (Electrical Geodesics, Inc.) and the examined regions of interest.** The electrodes included in the parietal ROI of the conflict slow potential (cSP) are marked in blue. Electrodes included in the fronto-central ROI of the N1 and N2 are gray.

During the experiments, continuous EEG was recorded with an Electrical Geodesics Inc (2016) 128-channel system (see **Figure 2** for a schematic illustration of the electrode net) with a sampling rate of 500 Hz and Cz as the reference electrode (Electrical Geodesics Inc, 2016; EGI, Eugene, OR, USA; Tucker, 1993). Impedance was monitored throughout the recording and kept below 50 kΩ. Further processing steps were performed with BrainVision Analyzer 2.0 (Brain Products GmbH, Gilching, Germany).

After visual inspection of the data, the continuous EEG was filtered (low cutoff: 0.5 Hz, time constant: 0.3, 12 dB/Oct; high cutoff: 40 Hz, 12 dB/Oct; notch filter: 50 Hz) and EOG artifacts were removed by semiautomatic ocular correction, using an ICA algorithm as implemented in BrainVision Analyzer 2.0 (Slope Mean, over the whole data, ICA with infomax algorithm, total squared correlations to delete: 30%; Gratton et al., 1983; Plank, 2013). Other artifacts were excluded automatically (gradient criteria: more than 50 μV difference between two successive data points or more than 100 μV difference in a 100 ms window; absolute amplitude criteria: amplitudes exceeding +150 or -150 μV; low activity criterion: less than 0.5 μV activity in a 100 ms window) and the EEG was re-referenced to the average of the mastoids.

The data was then segmented into epochs from -200 to 1000 ms relative to stimulus onset. The 200 ms pre-stimulus period was used for baseline correction. Afterward, the individual ERPs were averaged separately for each experimental condition and each participant group. Only correct trials were analyzed. In order to be included into the final analysis, participants had to have a minimum of 30 correct, artifact-free trials in each experimental condition. The average number (M [SD]) of accepted trials for the control group was 56 [2.53] and 57 [2.55] in the letter-speech sound interference experiment and 55 [4.01] and 56 [3.42] in the visual interference experiment (incongruent and congruent condition, respectively). For the dyslexic group, there were on average 54 [3.95] and 55 [4.29] accepted trials in the letter-speech sound interference experiment and on average 53 [2.99] and 55 [3.04] accepted trials in the visual interference experiment (incongruent and congruent condition, respectively). Based on non-parametric Mann–Whitney-*U*-test, the difference between the control and the dyslexic group in the average number of accepted trials was significant (all *p*s = 0.012–0.048). The number of accepted trials was somewhat higher in the control than in the dyslexic group. However, please note that the average number of accepted trials is at a very high level in both groups, consistently above 53 out of a maximum of 60 (corresponding to 89%), which can be considered as being close to ceiling.

Based on previous conflict-related ERP studies, we expected to observe the biggest N1 and N2 amplitude differences over frontal sites. The visual inspection of the data confirmed this assumption, thus, we defined our region of interest (ROI) over frontal sites, including the electrodes 3, 4, 5, 6, 10, 11, 12, 16, 18, 19, 20, 23, 24, 118, and 124 (see **Figure 2**). We searched for the most negative peak (local maximum) in the time window between 70 and 140 ms for the N1 and in the time window between 280 and 380 for the N2. Mean peak amplitudes and latencies were exported for each electrode of the above defined frontal ROI. The cSP is commonly observed over parietal regions, which was confirmed by visual inspection of our data, thus we defined a parietal ROI including the electrodes 61, 62, 67, 72, 77, and 78 (see **Figure 2**). For statistical analysis of the cSP, we exported the mean amplitude value for each electrode of the ROI between 500 and 900 ms. After the above defined exportations, the values of individual peak amplitudes, latencies and mean values were averaged over the electrodes included in the frontal and parietal ROI, respectively.

As there is evidence that cortical activation in letter-processing tasks might be less left lateralized in children with dyslexia (Moll et al., 2016), we considered the inclusion of the factor laterality in our analysis by building separate ROIs in the left and right hemisphere. In order to examine possible laterality differences between the groups, we conducted exploratory analysis comparing N1 and N2 amplitudes between the groups at different frontal locations (left, right, and central side). We found no laterality by group interaction effects (*p* > 0.19 and *p* > 0.36 for the N1 and N2 amplitudes, respectively), thus we did not consider laterality in further analysis.

### Statistical Analysis

Before statistical analysis of the behavioral data, RTs were outlier-corrected in two steps. In the first step, based on the distribution of all RTs across all participants, extreme values below 200 ms and above 10,000 ms were excluded. Afterward, RTs deviating more than 3 SD from the individual mean of each subject were removed. This processing resulted in the exclusion of 2.13% of the RTs in the letter-speech sound interference and the exclusion of 2.12% of the RTs in the visual interference task. Only correct answers were analyzed.

For the analysis of both the RT and EEG data, we computed mixed-model repeated measures ANOVAs including the within-subject factor *congruency* (congruent vs. incongruent condition) and the between-subject factor *group* (control group vs. dyslexic group) with an alpha level of 0.05. Significant interactions involving the factor group were examined with two-sided *post hoc t*-test.

The reliability of our novel paradigm was assessed using split-half correlations (Pearson’s coefficient; two-sided) of the mean RTs of the individual conditions. Correlation between the mean RTs of the first half and second half of the trials was consistently high in every condition; *r* = 0.91, *p* < 0.001 for the incongruent and *r* = 0.91, *p* < 0.001 for the congruent condition in the letter-speech sound interference task, and *r* = 0.85, *p* < 0.001 for the incongruent and *r* = 0.91, *p* < 0.001 for the congruent condition in the visual interference task.

## Results

### Behavioral Data

#### Error Rates

Error rates were very low in both the control and the dyslexic group. In the control group, the average error rate was 3.78% [3.13] and 0.95% [1.28] in the letter-speech sound interference experiment and 4.59% [3.82] and 1.98% [2.60] in the visual interference experiment (incongruent and congruent condition, respectively). For the dyslexic group, there was an average error rate of 6.02 and 3.52% in the letter-speech sound interference experiment and an average error rate of 6.90% [4.61] and 3.52% [3.42] in the visual interference experiment (incongruent and congruent condition, respectively). These high accuracy levels can be considered as being at ceiling. This might explain that even though the actual group difference between the error rates was very small, it still resulted in a significant main effect of group both in the letter-speech sound interference, *F*_(1,71)_ = 11.92, *p* < 0.01; $\eta_{p}^{2}$ = 0.14 and in the visual interference experiment, *F*_(1,71)_ = 7.55, *p* < 0.01; $\eta_{p}^{2}$ = 0.10.

We further found a strong congruency effect in both, the letter-speech sound interference, *F*_(1,71)_ = 35.27, *p* < 0.001; $\eta_{p}^{2}$ = 0.33, and the visual interference experiment, *F*_(1,71)_ = 35.33, *p* < 0.001; $\eta_{p}^{2}$ = 0.33. Accuracy rates were higher in the congruent than in the incongruent condition in both experiments. There was no significant interaction between congruency and group, neither in the letter-speech sound interference (*p* = 0.71), nor in the visual interference experiment (*p* = 0.45).

The average error rate in the “yes”-answer condition was 3.63% [3.21] and 5.62% [4.29] in the control group, and 5.22% [3.14] and 8.49% [5.17] in the dyslexic group (for the letter-speech sound interference and visual interference experiment, respectively).

#### Response Times

**FIGURE 3:**
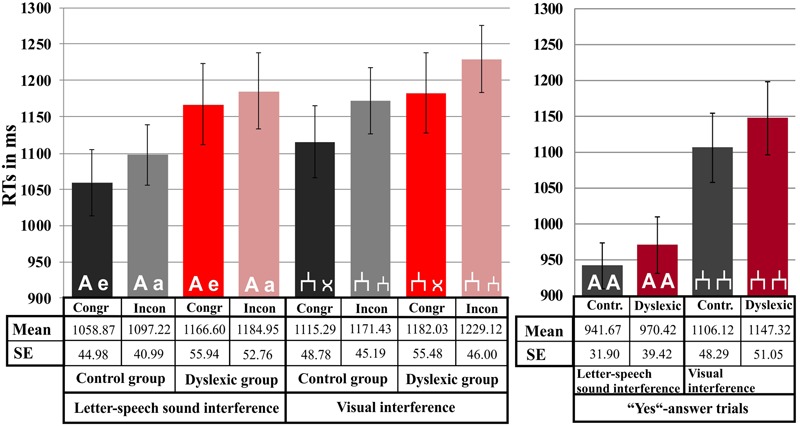
**Response time results.** Mean values are depicted separately for each experiment, condition and group. Error bars represent the standard error of mean.

There was no RT difference between the groups neither in the letter-speech sound interference, *F*_(1,71)_ = 2.04, *p* = 0.16, nor in the visual interference experiment, *F*_(1,71)_ = 0.84, *p* = 0.36. However, there was a strong congruency effect in both the letter-speech sound interference, *F*_(1,71)_ = 7.86, *p* < 0.01; $\eta_{p}^{2}$ = 0.10, and the visual interference task, *F*_(1,71)_ = 13.12, *p* < 0.01; $\eta_{p}^{2}$ = 0.16. RTs were faster in the congruent than in the incongruent condition in both experiments (see **Figure 3**). There was no significant interaction between congruency and group, neither in the letter-speech sound interference (*p* = 0.33), nor in the visual interference experiment (*p* = 0.75).

### ERP Data

**FIGURE 4:**
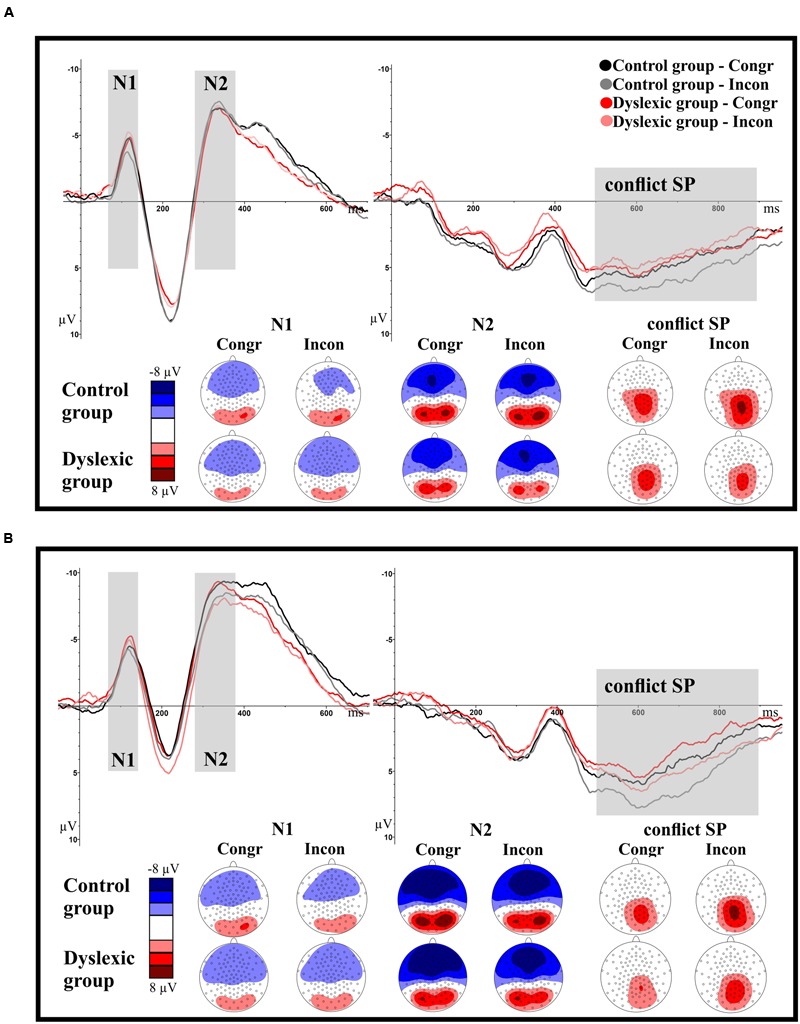
**Illustration of the grand-average ERP waveforms depicted separately for the fronto-central (N1, N2) and the parietal ROIs (cSP) and the letter-speech sound interference and visual interference experiments.** The time windows selected for the components N1 (70–140 ms), N2 (280–380 ms) and cSP (500–900 ms) are highlighted in gray. Negativity is depicted upward. **(A)** ERP components (averaged per group and condition) in the letter-speech sound interference experiment. **(B)** ERP components (averaged per group and condition) in the visual interference experiment.

Event-related potential waveforms including the N1, N2, and cSP components are depicted in **Figure 4**, separately for the letter-speech sound interference and visual interference experiments. Group mean of amplitudes and latencies are reported in **Table 2**.

**Table 2:** Means of peak amplitudes and latencies reported separately for the groups and conditions.

		Control group		Dyslexic group	
		Incon	Congr	Incon	Congr
**Letter-speech sound interference**
N1	Amplitude	-3.38 (2.92)	-4.56 (3.43)	-4.63 (3.03)	-4.15 (3.06)
	Latency	111.37 (9.42)	112.62 (10.65)	113.94 (9.53)	115.37 (9.11)
N2	Amplitude	-8.01 (5.97)	-7.74 (5.37)	-7.51 (5.37)	-7.34 (5.22)
	Latency	337.17 (18.59)	334.97 (19.81)	336.40 (14.87)	337.15 (16.60)
cSP	Mean amplitude	5.97 (5.08)	4.85 (4.51)	4.05 (4.31)	4.59 (3.94)
**Visual interference**
N1	Amplitude	-4.17 (3.47)	-4.18 (3.20)	-4.33 (3.00)	-4.78 (3.69)
	Latency	112.75 (12.43)	114.08 (10.77)	116.69 (7.51)	116.43 (10.04)
N2	Amplitude	-9.10 (7.11)	-9.91 (7.53)	-8.37 (5.59)	-9.71 (5.47)
	Latency	340.52 (16.80)	337.68 (16.93)	341.84 (15.99)	337.74 (18.33)
cSP	Mean amplitude	5.82 (7.23)	4.15 (5.32)	4.72 (3.32)	3.34 (3.92)

*Amplitudes are measured in μV, latencies in ms. Standard deviations are reported in brackets.*

### Letter-Speech Sound Interference Experiment

#### N1 Amplitudes and Latency

We found no main effect of group or congruency with respect to N1 amplitudes (all *p*s > 0.34). However, there was a significant group by congruency interaction, *F*_(1,71)_ = 5.42, *p* = 0.02; $\eta_{p}^{2}$ = 0.07. *Post hoc t*-test between the conditions revealed a significant difference between congruent and incongruent trials in the control group, *t*(_36_) = 2.12, *p* = 0.04; $\eta_{p}^{2}$ = 0.11. Amplitudes were less negative in the incongruent than in the congruent condition. There was no significant difference between the conditions in the dyslexic group, *t*(_35_) = 1.09, *p* = 0.28; $\eta_{p}^{2}$ = 0.03.

There were no significant main effects or interaction with respect to N1 latencies (all *p*s > 0.15).

#### N2 Amplitudes and Latency

There were no significant main effects or interactions with respect to N2 amplitudes and latencies (all *p*s > 0.40).

#### Conflict SP Mean Amplitudes

We found no main effect of group or congruency (all *p*s > 0.27) but a significant group by congruency interaction, *F*_(1,71)_ = 4.39, *p* = 0.04; $\eta_{p}^{2}$ = 0.06. The difference between the congruency conditions approached significance in the control group, *t*_(36)_ = 2.01, *p* = 0.05; $\eta_{p}^{2}$ = 0.10. Mean amplitudes tended to be higher in the incongruent compared to the congruent condition. There was no significant difference between the conditions in the dyslexic group, *t*_(35)_ = 0.96, *p* = 0.35; $\eta_{p}^{2}$ = 0.03.

### Visual Interference Experiment

#### N1 Amplitudes and Latency

There were no significant main effects or interactions with respect to N1 amplitudes and latencies (all *p*s > 0.12).

#### N2 Amplitudes and Latency

There was a main effect of congruency with respect to N2 amplitudes, *F*_(1,71)_ = 4.97, *p* = 0.03; $\eta_{p}^{2}$ = 0.07, but no other significant effect or interaction (all *p*s > 0.58). N2 amplitudes were less negative in the incongruent than in the congruent condition.

There were no significant main effects or interaction with respect to N2 latencies (all *p*s > 0.10).

#### Conflict SP Mean Amplitudes

We found a significant main effect of congruency, *F*_(1,71)_ = 10.33, *p* < 0.001; $\eta_{p}^{2}$ = 0.13. Mean amplitudes were higher in the incongruent than in the congruent condition. There was no other significant effect or interaction (all *p*s > 0.40).

## Discussion

The aim of the present study was to investigate the degree of automatization in letter-speech sound processing in children with dyslexia compared to TD children. For this reason, we implemented a newly designed letter-speech sound interference experiment and a visual interference control experiment while recording EEGs. In the letter-speech sound interference experiment, children were presented with two letters, and had to decide whether the two letters looked visually the same. In the incongruent condition, where the same letter was presented twice, once in upper and once in lower case (e.g., A-a), we expected to find a conflict between the visual information (the visual characteristics of A-a) and the automatically activated phonological information (both letters activate the sound /a/). The size of this conflict was supposed to reflect letter-speech sound automatization strength. In the congruent condition (e.g., A-e) in turn, no conflict was expected. In the visual interference experiment, the conflict was based purely on the visual similarity of the stimuli, thus this experiment served as a control experiment. We were interested in four questions which we are going to discuss now: (1–2) Are there any differences between children with dyslexia and TD children in the automatization degree of letter-speech sound processing and in the temporal sequence of letter-speech sound association processes? These questions are discussed in the section letter-speech sound interference experiment. (3–4) Do children with dyslexia have impairments in visual attention and visual conflict processing, and if so, are these impairments general or restricted to letter processing involving visual-verbal access? These questions are discussed in the section visual interference experiment.

### Letter-Speech Sound Interference Experiment – Measuring the Automatization Strength of Letter-Speech Sound Associations

We found a strong interference effect, reflected in the RTs of all participants. As expected, RTs were slower in the incongruent than in the congruent condition. Thus, the experimental manipulation worked as intended: speech sounds associated with the presented letters got automatically activated, which resulted in a conflict between the task-relevant and the task-irrelevant information as evident by slowed-down RTs. Furthermore, in the TD control group we found less negative N1 and more positive cSP amplitudes in incongruent compared to congruent trials. However, there was no difference between the N2 amplitude heights of incongruent and congruent trials.

The fronto-central N1, measured in our experiment 70–140 ms after stimulus presentation, is related to conflict sensory detection (Yu et al., 2015). As N1 amplitudes were modified in the control group, we conclude that the conflict between the phonological (speech sound) and visual information emerged in the control group in a very early time window within the first 140 ms. This implies that in TD 9-year-olds, letters activated their corresponding speech sounds in a highly automatic manner, almost immediately. The direction of the conflict-related N1 amplitude modification was thereby the same as in previous studies using an auditory Stroop-paradigm: N1 amplitudes were less negative in incongruent than in congruent trials (Lew et al., 1997; Henkin et al., 2010; Yu et al., 2015). This similarity to the findings of auditory paradigms reinforces the assumption that the conflict in our letter-speech sound interference paradigm was really based on the automatic activation of phonological information.

Importantly, we found no evidence of conflict-related N1 amplitude effects in the dyslexic group. Thus we assume that in children with dyslexia speech sounds associated with the letters were not activated at this early time point (140 ms after stimulus presentation).

The parietal cSP, measured in our study 500–900 ms after stimulus presentation, is linked to conflict resolution and response selection processes (Yu et al., 2015). As expected, cSP amplitudes were higher in the incongruent than in the congruent condition within the TD group (Donohue et al., 2012; Yu et al., 2015). However, there was again no evidence of conflict-related amplitude effects in the group of children with dyslexia. Thus, it seems that speech sounds associated with letters did not get automatically activated until 900 ms after stimulus onset in 9-year-old children with dyslexia.

However, children with dyslexia showed congruency effects in the behavioral measure. In both groups, RTs were slower in the incongruent than in the congruent condition. Thus, as there was a behavioral effect of conflict in children with dyslexia, speech sounds must have been activated at some point in time, most probably after 900 ms of letter presentation. This finding is in line with previous electrophysiological investigations: electrophysiological studies implementing an audiovisual MMN paradigm have found delayed letter-speech sound association effects in children with dyslexia compared to TD children (Froyen et al., 2011; Moll et al., 2016). As activation of speech sounds was not necessary for solving the letter-speech sound interference task, the delayed association observed in the ERP data might not have hampered task performance, which might explain why there was no overall RT difference between the groups. However, delayed letter-speech sound association might impact on RTs in reading related tasks where letter-speech sound association are consistently required during task performance.

Finally, we need to discuss the lack of a conflict-related N2 effect. Based on the findings of Yu et al. (2015) we expected to find less negative N2 amplitudes in incongruent compared to congruent trials, even though findings on conflict-related N2 amplitude modifications are still very mixed: there are studies reporting enhanced conflict-related N2 amplitudes (van Veen and Carter, 2002a,b; Yeung et al., 2004; Johnstone et al., 2009), or a null result (Henkin et al., 2010). We have two possible explanations, which might account for the different findings: first, we have to consider the differences between the designs of the two paradigms: in the study of Yu et al. (2015) the stimuli were spoken Chinese words, presented auditory. In our study, however, we displayed single letters and false fonts, visually. As proposed in the three stages cognitive control model for auditory Stroop task (Yu et al., 2015), the N2 reflects the conflict identification stage, including the categorization and coding of the conflict information. As our letter-stimuli were quite simple, it might be possible that no categorization was needed or that the categorization process was faster than in other paradigms and thus, already fully accomplished at the time point of the N2. A second possible explanation may be that there was a big difference between the age of our participants and the age of the participants in Yu et al.’s (2015) study. We examined 9-year-old children, whereas the participants of Yu et al. (2015) were healthy students. As electrophysiological studies investigating auditory Stroop-paradigms in children are missing, we cannot rule out that conflict-related N2 amplitude effects change with age. In order to examine this possible explanation, further studies comparing different age groups are needed.

Taken together, the letter-speech sound interference paradigm is useful to measure automatization strength of letter-speech sound associations. The experimental manipulation resulted in strong behavioral effects in both groups and fast and strong neurophysiological correlates in the control group. Furthermore, we revealed neurophysiological differences between TD children and children with dyslexia in the automatization strength of letter-speech sound associations in a relatively naturalistic setting of sole visual letter presentations. Speech sounds associated with letters were activated very fast in TD children; the first effects being present already within 140 ms. In contrast, children with dyslexia did not show neurophysiological evidence of automatic letter-speech sound activation effects. Thus, we can conclude that letter-speech sound associations are highly automatic in TD 9-year-olds, but are less automatized in children with dyslexia.

### Visual Interference Experiment – Measuring Visually Based Conflict Processing

Response times were slower in the incongruent than in the congruent condition, thus there was a strong interference effect. Also, N2 amplitudes were less negative in incongruent than in congruent trials, and cSP amplitudes were more positive in incongruent than in congruent trials. Thus, we can conclude that the experimental manipulation successfully induced conflict. However, there was no difference between the N1 amplitudes of incongruent and congruent trials, and there was no difference between the groups in the behavioral and ERP-effects of conflict.

The finding of increased cSP amplitudes in incongruent trials matches the results of existing studies (Liotti et al., 2000; Larson et al., 2009). However, the finding of less negative N2 amplitudes in incongruent trials compared to congruent trials is contradictory to previous findings. Neurophysiological studies implementing the flanker task reported more negative N2 amplitudes in incongruent than in congruent trials (Yeung et al., 2004; Johnstone et al., 2009).

The reason for this discrepancy between our finding and the findings of the flanker task studies might be that the flanker task – although it is implemented in order to induce visual and response conflict, which makes it in some ways comparable to our study – has a completely different design than our study. It uses flanker-stimuli to induce conflict (e.g., > > > > > or > > < > >) whereas we presented two false fonts side by side. Thus, the conflict of the flanker task is determined by the suppression of flankers in the periphery, whereas our conflict is based solely on visual similarity. Another explanation might be the difference between the paradigms in their definition of congruency. In our visual interference experiment, congruent trials are defined as congruent, because of an overarching dimension of congruency; the false fonts look “different,” and the required answer is also “different.” In contrast, the congruency in the flanker task experiments is based on the similarity of the target stimulus to the flankers. Thus, our congruent condition might be probably stronger comparable with the incongruent condition of the flanker task, where flankers are also perceptually and categorically different from their targets. As our study is the first to use this design, further studies are needed to clarify this issue. Based on our findings, we assume that visual conflict, implemented in the way as in our visual interference experiment (i.e., based purely on visual similarity) results in diminished N2 amplitudes. This assumption is supported by the fact that the directionality of the conflict related N2 amplitude modification might change with the properties of the presented stimuli (e.g., less negative in Yu et al., 2015; more negative in Johnstone et al., 2009).

Importantly, there were no differences between the groups in the direction and extent of the conflict-related behavioral N2 and cSP effects. Visual conflict resulted in longer RTs, diminished N2 and increased cSP amplitudes in both groups. These findings differ from the results of Mahé et al. (2014), who found impaired conflict monitoring and conflict resolution processes in a group of dyslexic adults, reflected in reversed N1 and missing N2 and P3b effects when compared to controls. One possible explanation for the discrepant findings could be that subclinical attentional deficits were not considered in the (Mahé et al., 2014) study. Even though they excluded participants with a diagnosis of ADHD, they did not assess attentional problems in their participants. Individuals with dyslexia often show subclinical problems of inattention, hyperactivity and impulsivity, even though they might not fulfill diagnostic criteria for ADHD (Smith and Adams, 2006). In order to control for subclinical problems of inattention, hyperactivity and impulsivity we explicitly assessed those symptoms. Importantly, our groups did not differ with respect to these symptoms. Another explanation for the discrepant findings is that, as already discussed above for the N2, there was a huge difference between the design of our and Mahé et al.’s (2014) experiment. Mahé et al. (2014) implemented a flanker task, whereas we displayed false fonts. The conflict of the flanker task is induced by the need for flanker suppression, and thus implies the automatic shifting of attention toward stimuli according to the congruency of flankers. For this reason, the findings of Mahé et al. (2014) can mainly be explained by difficulties of the dyslexic group in attentional shifting and flanker suppression. Our study, in contrast, measured visual conflict processing on a more general level based on sole visual similarity without attention shifting. Thus, based on our results we conclude that visual conflict processing is not impaired in dyslexia. However, we cannot exclude that children with dyslexia might have impairments in attentional shifting and suppression of flankers. This interpretation is similar to the conclusions of Bednarek et al. (2004), who also reasoned that TD children and children with dyslexia did not differ in their general perceptual and attentional abilities but are impaired in specific domains, such as narrowing the focus of attention and the inhibition of flanker interference. However, as our study is among the first electrophysiological investigations on conflict control processing in dyslexia, findings in this domain are still very rare. In order to reinforce the above assumption, further investigations are needed.

Finally, we would like to discuss the lack of conflict related N1 effects, which contradicts previous findings. Visual conflict studies implementing the flanker task reported increased N1 amplitudes in incongruent compared to congruent trials (Yeung et al., 2004; Johnstone et al., 2009). We assume that the discrepancy can be explained by methodological differences. False fonts are unknown and visually more complex than the arrows in the flanker task. Thus, visual processing and conflict detection are likely to be slower in the visual interference task than in the flanker task. This is likely to result in a delay of the conflict related effects, resulting in conflict-related modulations in the N2 but not in the N1 amplitude.

To summarize, the results of the visual interference experiment suggest no visual conflict processing deficits in dyslexia. Visual conflict had comparable behavioral and neurophysiological effects in children with dyslexia and TD children. However, studies examining the neurophysiological underpinnings of conflict processing in dyslexia are still very rare, thus our findings need to be replicated.

### Limitations

Our study sample was very homogenous, consisting of mostly 9- and 10-year-old children (age range: 8.42–11.25). Although a homogenous sample increases the power of statistical tests, it has a negative impact on the generalizability of the results. It is important to consider age-related differences, especially when studying developmental disorders. It might be possible that the development of automatized letter-speech sound associations is only delayed but not permanently impaired in children with dyslexia. Thus, further longitudinal studies examining the neurophysiological development of letter-speech sound association in dyslexia are needed.

It must be considered that our participants were all German-speaking. As the German language is characterized as a shallow orthography with very consistent letter-speech sound correspondences, it is possible that the effects of our letter-speech sound interference paradigm are stronger in this population. In deep orthographies, as for example in English, letter-speech sound associations might not always be unambiguous. Especially vowels are often pronounced in various ways in English. Thus, for the implementation of the letter-speech sound interference paradigm in English-speaking populations, the application of consonants might be more suitable. This assumption is strengthened by the findings of Posner and Mitchell (1967), who found that RTs indicating the physical identity of the objects were slowed by the same name only for the letter-pairs B-b and C-c, but not for the letter-pairs A-a and E-e, although this finding has already been challenged (Carrasco et al., 1988). Cross-linguistic studies might help to clarify the question of generalizability of these findings.

## Conclusion

The letter-speech sound interference paradigm has proved to be a good neurophysiological paradigm to examine automatization strength of letter-speech sound associations in TD children and children with developmental dyslexia. Our results point to highly automatic letter-speech sound associations in TD 9-year-old children, whereas letter-speech sound associations seem to be less automatic in children with dyslexia.

The visual interference paradigm extended neurophysiological findings on visual conflict processing in developmental dyslexia. Children with dyslexia and TD children were comparable in the neurophysiological and behavioral visual incongruency effects, thus, we did not confirm the assumption of a general impairment in conflict control processing in dyslexia.

## Author Contributions

KL, GS-K, and KM were involved in the conception and design of the study. SB, JB, GS-K, and KM developed the methods. SB and KM acquired the data. SB, JB, and KM performed the analyses. SB and KM wrote the first version of the manuscript. All authors commented on the manuscript and approved the final version of the manuscript.

## Conflict of Interest Statement

The authors declare that the research was conducted in the absence of any commercial or financial relationships that could be construed as a potential conflict of interest.
